# Participants’ views and experiences from setting up a shared patient portal for primary and specialist health services- a qualitative study

**DOI:** 10.1186/s12913-021-06188-8

**Published:** 2021-02-24

**Authors:** Torunn Hatlen Nøst, Arild Faxvaag, Aslak Steinsbekk

**Affiliations:** 1 Department of Mental Health, Faculty of Medicine and Health Sciences, Norwegian University of Science and Technology, 7491 Trondheim, Norway; 2 Department of Neuromedicine and Movement Science, Faculty of Medicine and Health Sciences, Norwegian University of Science and Technology, Trondheim, Norway; 3 Clinic of Orthopaedy, Rheumatology and Dermatology, St. Olavs Hospital, Trondheim University Hospital, Trondheim, Norway; 4 Department of Public Health and Nursing, Faculty of Medicine and Health Sciences, Norwegian University of Science and Technology, Trondheim, Norway; 5 Digital Health Care Unit, Norwegian Centre for E-Health Research, Tromsø, Norway

**Keywords:** Patient portal, eHealth, Configuration, Qualitative

## Abstract

**Background:**

Recently, there has been an increasing focus among healthcare organisations on implementing patient portals. Previous studies have mainly focussed on the experiences of patient portal use. Few have investigated the processes of deciding what content and features to make available, in particular for shared portals across healthcare domains. The aim of the study was to investigate views on content and experiences from the configuration process among participants involved in setting up a shared patient portal for primary and specialist health services.

**Methods:**

A qualitative study including 15 semi-structured interviews with persons participating in patient portal configuration was conducted from October 2019 to June 2020.

**Results:**

Whether a shared patient portal for all the health services in the region should be established was not questioned by any of the informants. It was experienced as a good thing to have numerous participants present in the discussions on configuration, but it also was said to increase the complexity of the work. The informants considered a patient portal to be of great value for patient care, among other things because it would lead to improvements in patient follow-up and increased patient empowerment. Nevertheless, some informants advocated caution as they thought the patient portal possibly could lead to an increase in healthcare providers’ workloads and to anxiety and worries, as well as to inequality in access to health care among patients. The findings were categorized into the themes ‘A tool for increased patient involvement’, ‘Which information should be available for the patient’, ‘Concerns about increased workload’, ‘Too complex to use versus not interesting enough’, ‘Involving all services’ and ‘Patient involvement’.

**Conclusions:**

Establishing a shared patient portal for primary and specialist health services was considered unproblematic. There was, however, variation in opinions on which content and features to include. This variation was related to concerns about increasing the workload for health care providers, causing anxiety and inequality among patients, and ensuring that the solution would be interesting enough to adopt.

## Background

The delivery of knowledge- and value-based care depends on patients taking an active role in their caregiving [1, 2]. In many countries, this knowledge has translated into health policies and legal frameworks where a common objective is to transform health systems by increasingly putting the patient at the centre of care and empowering patients to take an active role in their health and health care by the use of technology [3, 4]. As part of this process, there has been a growing focus among health care organisations on implementing patient portals as a means to provide patients access to their electronic health record (EHR) data [5–8].

Patient portal features range from viewing health information in the EHR such as discharge summaries and medication lists to features enabling patients to make requests, add data, interact with healthcare providers and view individualised educational materials [9]. Although the potential of patient portals to empower patients is widely agreed on, systematic reviews on the effects and impacts of patient portals vary from showing some effects to noting clinically non-relevant effects [10]. Moreover, it has been stated that the patient portals have not reached their expected potential and issues such as patients’ privacy concerns, lack of endorsement and encouragement from providers, features perceived as being inadequate, varying usability of the portals, and personal factors among both patients and providers have been suggested as possible explanations [8, 9]. To overcome the mentioned issues, participation of the involved healthcare organisations’ staff and patients are emphasised as important to ensure that the configurated patient portal solutions align with the needs expressed by future users [9, 11].

The majority of studies on patient portals are from the US, which means that there is less knowledge on patient portals in a European context [12–14]. Most studies are conducted within one healthcare domain [15, 16] or on patient portals for specific conditions [17, 18]. Although eHealth tools have been put forward as central for improving integrated care [19, 20] few studies have focused on the processes of establishing a patient portal across healthcare domains [21]. Furthermore, we have not found any studies that investigate experiences of setting up a patient portal for services across healthcare domains.

Hence, the aim of the study was to investigate views on content and experiences of the configuration process among participants involved in setting up a shared patient portal for primary and specialist health services.

## Methods

A qualitative study with semi-structured interviews including participants involved in a patient portal configuration process was conducted from October 2019 to June 2020.

### Setting

The study was conducted in an ongoing project for procurement and implementation of an EHR solution for all the health services in Central Norway, a region with approximately 720,000 citizens and 40,000 healthcare professionals, including hospitals, general practitioners, nursing homes, public health centres and home-care services (www.helseplattformen.no). The project aims to give the citizens an easy access to their medical records and an opportunity to influence their own course of treatment, as well as to contribute to the objectives in the Norwegian eHealth policy on ‘One citizen- One record’, where it is stated that citizens shall have access to user-friendly and secure digital health services, updated health information and individual patient-centric health plans [22, 23].

The project has chosen Epic Systems Corporation (https://www.epic.com/) with the patient portal MyChart as their solution. MyChart is a web-based patient portal that gives patients access to the same EHR used by their healthcare providers. MyChart includes features that allow patients to communicate with their healthcare providers, request prescription refills, manage appointments and access test results and providers’ notes. Which features will be made available for the current project, is being discussed during the ongoing configuration work and was not yet decided at the point of this study’s data collection.

The informants were connected to the configuration work of the patient portal. This work is organized the same as other areas in the project, where stakeholders representing the different involved organisations and services participate in a series of workgroup sessions. The work with the patient portal began in early autumn 2019 with direction-setting sessions on the topics ‘activation and adoption’, ‘patient interaction and patient engagement’, and ‘video visits and telehealth workflows’. This was followed by a phase on content sessions during the spring of 2020, including sessions on goal direction, workshops on guiding principles and sessions on adoption. The work will proceed with iterative activities on building and testing the solution before implementation.

### Informants and recruitment

Eligible informants were persons involved in the project’s patient portal work, such as healthcare providers appointed to represent their clinical field of expertise, representatives from the vendor with relevant experiences from other countries, patient representatives participating in the patient portal workgroup and employees in the project engaged with the patient portal. To obtain data that represented variations in experiences from participating in the patient portal work, the aim was to have variation in roles in the project and in the home organisations for main employee positions.

The first author recruited the informants based on information from the project management on who had participated in the patient portal work. The persons who were identified as meeting the inclusion criteria were sent an invitation from the first author that included information on the intentions of the study and a consent form. All those invited agreed to participate. Recruitment continued until 15 informants were interviewed and the data collected were considered sufficient to answer the study’s aim [24].

### Data collection and interview guide

Data were collected during a period where the patient portal workgroup was engaged in direction-setting sessions and content sessions, that is, in the early phases of the configuration process. The first author conducted all the interviews. Based on the choice of the informant and the Covid-19 regulations, six of the interviews were done using a digital platform. The other interviews were conducted either at the premises of the project or at the informants’ workplaces. All interviews were audio recorded and transcribed verbatim. The interviews lasted between 25 and 75 min (mean duration 53 min).

The semi-structured interview guide was developed based on the study aim, literature in the area of patient portals and discussions among the authors. The main question asked was how they had experienced the discussions and the work on the patient portal so far. If the topics were not spontaneously talked about, they were also asked how they considered a patient portal to benefit the patients and the health services and whether there were specific discussions that they had found challenging.

### Data analysis

The data were analysed using systematic text condensation, which is a descriptive thematic cross-case analysis strategy involving an iterative four-step analysis procedure [25]. First, the authors worked to gain an overall impression of the data by reading the interviews. As a starting point, all authors read the same three transcripts, resulting in the identification of five preliminary main themes answering the aim. Thereafter, the first author systematically reviewed all the interviews to identify meaning units relevant to the aim. The meaning units were coded, classified and sorted into code groups related to the preliminary themes, and these were repeatedly discussed among the authors. During this part, the themes were changed from focusing on the timeline of the project to focusing more on the issues the informants were most concerned about. In the third step, the first author performed a systematic abstraction of meaning units within each of the themes, reducing the content into a condensate that maintained the informants’ responses. The authors had repeated discussions on the condensates, resulting in adjustments and renaming of the themes. In the final step, the content of the condensates was synthesised into generalised descriptions and concepts used in the result section, while ensuring that the result still reflected the original context.

The first author identified illustrative citations, which were translated by the first author and validated by the co-authors. To preserve the anonymity of the informants, it was chosen not to mark who the citations were from. MindManager [26] was used as the systematisation tool during the analyses. To expose the analysis to different views and perspectives, preliminary results were discussed both with an extended research group on patient education and participation that the first and last authors are members of and with researchers experienced within the field of digital health as well as presented to the management of the project.

## Results

In total, 15 persons were interviewed (Table 1). Few informants had prior experiences with a patient portal, and none of them had previously participated in establishing a shared patient portal for both primary and specialist health services.

**Table 1:** Characteristics of the informants

Characteristic	Number
Main employee position at:	
The EHR project (Helseplattformen AS)	3
The EHR vendor	4
The health trusts	3
The municipality, including GPs	3
Others	2
Role in the EHR project:	
Health service employee	6
Vendor’s representative	4
Helseplattformen employee	3
Patient representative	2

Whether a shared patient portal should be established for all the health services in the region was not questioned by any of the informants. They all considered the patient portal to be of great value for all parties involved in patient care, but some also raised critical issues regarding available content and features. The findings related to both the patient portal as an asset and areas where the patient portal could be a challenge were categorized into the themes ‘A tool for increased patient involvement’, ‘Which information should be available for the patient’, ‘Concerns about increased workload’, ‘Too complex to use versus not interesting enough’, ‘Involving all services’ and ‘Patient involvement’.

### A tool for increased patient involvement

The patient portal was viewed by the informants as one of the most important initiatives that the project would introduce, primarily because it would enable in an easy way the involvement of patients in information flow and decision making. This was in line with their view that including patients in care decisions was the right thing to do and, moreover, something that would increase the openness and transparency of the health services. This was perceived to build trust between the services and their patients. Some informants had from the start a stance of principle which was in line with the project’s overarching goal, namely that the patients should have easy access to their own EHR.

*We are very engaged with the openness issue. At the same time that it is- it is the patient’s record. It’s the patient’s record, the patient’s property. The patient is the one who can decide over it- decide what shall not be recorded, and if it should be recorded. The patient decides.*

Access to EHR information via the patient portal was expected to support patients in taking care of their own health and thus to strengthen the role of the patients. One of the reasons discussed was that the patient portal would give patients access to EHR information at all times without them having to ask or to be dependent on healthcare providers. In particular, the patient portal was regarded as beneficial not only for those who frequently utilised services across the healthcare system but also as a tool to reach the general public with information. For instance, low-threshold services such as public health centres were regarded to benefit from reaching the inhabitants of the municipality with public health information through a patient portal. Most of all, having a patient portal was perceived to make things easier and more efficient, both for patients and the health services.

*Simplicity. To get in touch in an easy way and understand what is going on. And get in touch with and all that and, yes, gain more control over your own health.*

### Which information should be available for the patient?

All informants wanted features such as accessing medication lists, requesting prescription refills, scheduling appointments and messaging, arguing that it would ease access to services and improve communication. However, when talking about what clinical information patients should have access to, the informants had different opinions. Some informants argued for limiting access to EHR information such as test results and notes before healthcare providers could assess and comment on them because it could cause anxiety and worry in patients.

*We feel very positively about patients having access to test results, but we would like them to have access to them after we have assessed the results and can give them guidance because we are very afraid that, for example, that patients will see the test results without us being able to give them help in understanding them.*

Other informants did not have these concerns. They argued that the patients should be the ones to decide whether and when they wanted to access the information. Informants mentioned examples such as patients with diabetes who were perceived to benefit from knowing their test results and cancer patients where available doctor notes after hospital visits could be useful to review at home together with family members. However, those arguing this position said that it had not been easy to come through with their arguments in the workgroup discussions. The reason was partly because views on more practical implications for the different professionals had dominated the discussions and partly because they felt that the idea of patients having access to EHR information still was relatively new and therefore not easy to bring into the discussions.

*There have been cases where the clinicians and partly the patient representatives have said that it should not be the default to share test results and medical records but that you* [the health care professional] *should actually actively say when you will share it. That is, in fact, the opposite of having openness and is, rather, hiding things.*

For some, the discussion on which content to make available in the patient portal was said to be an example of a conflict between the agreed-upon intentions of the patient portal and the healthcare providers’ willingness to change their practice. The first recommendation from the workgroup ended up being that healthcare providers should manually indicate which EHR data should be displayed for the patients and when. However, the leadership of the project sent the recommendation back for further discussion as it was perceived to not be in accordance with the project’s overarching goals.

*Yes, we had the decision sent back to us, to our group. And that’s fine because, as they say, it should be the patient who should be in focus … . The reason why it happened was that the basis for the meetings was insufficient. We were not properly informed that here we are only talking about Go-Live. It was sort of a question on do you want everything at once or not. It was kind of like that. And nothing in between.*

### Concerns about increased workload

One major concern repeatedly mentioned by some informants and referred to from the discussions in meetings by others was that the patient portal could lead to an increased workload for health professionals. It was used as an argument that features that could increase the workload should not be made available. One example discussed was that information released to the patient portal would require more follow-ups, which, for some services, meant they had to reorganise their workflows.

*And so, if the patient, for example, is to be able to add such things* [the patient’s own comments or notes in the EHR], *then someone must take a position on that information and confirm or deny it and do a job with the information that is entered. And I think that could lead to a- a potentially large workload for whoever is going to do that.*

Yet some informants considered that the patient portal would not necessarily mean more requests from patients but rather that the requests would be put forward on a new platform. For them, the issue was more to what extent the services were mature or agile enough to open up for more patient interaction through the patient portal. Some said that with time, this would change, but that for now, it was necessary to take one step at a time.

*We should decide on the principles. Do we want it this way or that way? Then there have been discussions on the number of clicks. There will be too many clicks, some say. So, yes, there are some small things that do not really mean that much where we have spent- we have lost some time on discussing such issues.*

### Too complex to use versus not interesting enough

Another concern among informants was that if too many features were to be implemented, the patient portal would become too complex to use. Some mentioned that the elderly would struggle to use it and that the patient portal could become most beneficial for those looked upon as most resourceful. Consequently, some were concerned that the patient portal could contribute to increased inequality in access to health care.

*It can be a risk that this will be- for those who- if you have the MyChart glasses on- that this will be for those who are the most resourceful in the first place and who can now order and arrange and such, while it will not be so good for those who are not so good at computers or do not understand how to use it.*

On the other hand, there were also concerns that the final product might not be interesting enough for people to actually use it. This could be due to both a lack of available information that is interesting to the patients and too little functionality. If this were to happen, informants said it would be because they as a workgroup had missed opportunities that would benefit patients in the future.

### Involving all services

It was repeatedly stated that it was important that the different services participated in the same set-up discussions. However, it was also said that, at times, it was difficult to give input as to the best solution for a shared patient portal because they knew little about the other involved services and they were not given time to get to know each other. Moreover, even though it was considered good to have numerous participants present in the discussions, it also was said to increase the complexity of the work because different needs and sometimes conflicting views made it harder to reach agreement.

*Maybe it will be easier in the long run, that you manage to see it as a whole- one health service, that we can work together on it. That is certainly not where we are now.*

Some of the informants explained that because they were appointed to represent their organisation in the patient portal workgroup, they felt an obligation to ensure that specific topics relevant for their service and profession were attended to in the discussions. However, they also experienced this as challenging when, for instance, arguments heavily based on how the solution would impact the different participants’ working situation kept the workgroup from focussing on what the best solution for the whole region would be.

*Because I think that up until now, many have experienced that it has largely been a discussion between GPs and hospital doctors. And they have strong voices.*

### Patient involvement

Most of the informants had wanted patient representatives to be part of the workgroup from the beginning because they assumed that they would bring other arguments to the discussions. It was said that having patient representatives present would remind them all of what the work really was about, and, as one of the patient representatives described it, they as patients could have a moderating effect on the discussions when the different services had conflicting interests. However, the plan for the project was to involve patient representatives in the workgroups later in the process.

*We have spent quite a lot of time on this with patient representation. That is, that we have missed having them in the group. So there have been several people really working hard on it, to get that in place.*

## Discussion

Informants in the current study did not express any concern regarding the establishment of a shared patient portal for the region’s health services. They focused most on the value they perceived it would have for patient care. There was, however, variation in opinions about which features that should be made available. This was related to arguments about the possibility of increasing the workload for healthcare providers, causing anxiety and worries as well as inequality among patients, and ensuring that the solution would be interesting enough to adopt. Some argued for making all features available regardless of such concerns, but their views did not prevail in, for instance, the first recommendation on release of test results and provider notes. The leadership of the project sent this recommendation back to the workgroup for further processing because it was not in line with the project’s overarching goal. The patient representatives did not participate in the workgroup from the beginning and the timing of patient involvement in the process was therefore questioned.

### No challenges with a shared patient portal?

Potentially, there could have been a disagreement among the informants on whether a shared patient portal would meet the needs of the different services. However, this was not the case. Even when asked directly, informants did not see any particular challenges to establishing a shared patient portal. Rather, it was argued for the value of a patient portal in general regardless of the number of or variations in services using it. The informants’ perception was thus in line with arguments that eHealth tools and patient portals have the potential to help improve care coordination, integrated care and management processes across services [19, 20].

Although establishing a shared patient portal was not perceived as problematic, discussions on the concrete content and features were at times experienced as challenging. This was most evident when some informants talked about what they considered a conflict between the agreed-upon intentions of a patient portal, for example, increasing patient empowerment, and arguments for limiting the features available due to concerns of possible negative consequences for both providers and patients. This was, in particular, related to discussions on which test results and provider notes should be available for patients and when. As it turned out, the leadership in the project turned down the workgroup’s first recommendation because it did not align with the project’s overarching goals on creating a patient-centred solution. As such, the management is in line with the OpenNotes initiative where transparency of information such as provider notes has been described as vital to enabling shared decision making and patient involvement [27].

### Does a patient portal lead to more work for the healthcare providers?

Several informants talked about their views on and the discussions in the meetings about the possibility of more work for the healthcare providers if certain features, for example, access for patients to see provider notes and comment on them, were activated. Whether patient portal use increases healthcare providers’ workload has been discussed and investigated by others as well [7]. Some studies have found the workload to increase for some [28], whereas other studies have found patient portal use to decrease workload [6]. A recent study on the clinicians’ experiences of sharing notes with their patients found that some subgroups of clinicians were less enthusiastic than others, but even among these, most of them endorsed the idea of sharing notes and believed it could be helpful for engaging patients more actively in their care [29]. Thus, there does not seem to be a single simple answer whether implementation of a patient portal will affect workloads. This is in line with the variation among the informants in the current study. Some argued that it would increase workload, but others did not, arguing that the number of requests would not increase but rather be moved onto a new platform where they could be handled more efficiently. As there is an association between provider’s engagement and patient portal adoption [9], the expressed concerns about increased workload can nevertheless be usefully addressed to increase the likelihood of a successful patient portal implementation.

### Can a patient portal have negative consequences for the patients?

The introduction of patient portals has been linked to patients’ empowerment, activation and involvement [30–32] and to contributing to a more active role for patients in decision-making, self-management of health conditions and coordination of health care [5–7]. Thus, patient portals can support patient-centred care [33]. Although such benefits were recognised in the study at hand, the informants were concerned whether the patient portal could lead to negative consequences both for the individual patient and for groups of patients.

For instance, some of the informants wanted to limit when and which information was made available to ensure that the information did not cause anxiety and worries among individual patients. Other studies have found both that patient portals can reduce patients’ anxiety within chronic care management [7] as well as create anxiety among admitted patients [8]. A recent Dutch study on real-time access to EHR information via a patient portal found some, yet few, examples of unwanted consequences with confused and anxious patients related to release of test results and clinical reports [34]. Similarly, a study on patients’ experiences of reading their clinicians’ notes reported that, overall, patients understood and found the notes useful, regardless of whether they were written by a general practitioner or a specialist [35]. This, and findings from other studies [13, 14, 36], supports the views of the informants in this study who were in favour of patient access to notes and test results, arguing that patients themselves know what is best for them and therefore should be the ones to decide whether and when they will access their EHR information. However, because the study was done in an early phase of the project, it is possible that, with time, the views on sharing information will change.

Another concern was about whether the patient portal would increase inequality, for example, that the patient portal would be most useful for the digitally competent and resourceful patients, especially if too many features were available so that it became complex to use. There are indications that persons with limited health literacy are less likely to use patient portals [37]. A recent review on the role of patient portals found that it can increase a digital divide between patients and emphasised the importance of addressing health equity when implementing and adopting patient portals [38]. However, none of these studies reported on a connection between equity and number of features but focussed more on the user-friendliness of the solutions.

### Patient representatives from the beginning

Including all involved stakeholders and partners in eHealth projects is highly recommended [39–41] because it can ensure that the designed solution corresponds to the needs expressed by its future users [9, 11]. Nevertheless, projects involving eHealth can be challenging due to issues such as introduction of new forms of cooperation and participation of a high number of stakeholders [3, 41]. In the current study, the informants talked about the value of patient representation to ensure that the end users arguments and perspectives were included in the discussions. This is in line with the literature on the usefulness and necessity of including patient representatives in health service development [42]. Nevertheless, the informants said that patient representatives were included after the work had begun and that this was related to the overall organisation of patient involvement in the project. Still, some informants found this difficult to understand, as their view was that patient representatives were especially important when setting up the patient portal as they advocated a perspective other did not necessarily have. However, for the later phases of the configuration the patient representatives were included on the same level as representatives from the healthcare services.

### Strengths and limitations

Strengths of the study are the novelty in exploration of experiences with the process of setting up a shared patient portal for primary and specialist health services, that informants had various previous experiences on the topic and that the interviews were conducted over time, which means that experiences from different perspectives and stages were covered. However, there are some noteworthy limitations. A main, but intended limitation, was that this study covered a limited part of the work with the patient portal and did not include the testing and building phase of the configuration or the actual use of the portal. The data collection was done in, and thus mirrors, an early phase of the configuration process. It is possible that at a later phase, the findings would have been different. Still, the data collection provided data to answer the study’s aim. The sampling strategy could have led to a biased sample as the informants were initially identified by the Helseplattformen’s management. Nevertheless, the sample showed variations as planned.

## Conclusion

This study on investigating views on content and experiences from the configuration process among participants involved in setting up a shared patient portal for primary and specialist health services found that establishing a shared patient portal solution was considered as unproblematic. There was, however, variation in opinions on which content and features to include. This variation was related to concerns about increasing the workload for healthcare providers, causing anxiety and worries as well as inequality among patients, and ensuring that the solution would be interesting enough to adopt. The insights provided by this study can inform implementation processes as well as policies on patient portals that include services across healthcare domains. The insights are of high relevance and importance due to the role of patient portals in supporting patient empowerment and patient-centred care. Furthermore, the results of the study regarding various concerns among the involved actors can be of value when preparing organisations for the implementation of a patient portal.

## Data Availability

The raw data supporting the findings of the manuscript can be found at the Department of Mental Health, Norwegian University of Science and Technology, Trondheim, Norway. Due to regulations of the Norwegian Social Science Data Services, NSD, the anonymity of the informants must be secured. In the raw data, it is possible to identify the informants, and restrictions therefore apply to the availability of these data. Reasonable requests concerning the data can be sent to the corresponding author.
